# Optimal cut-off points of anthropometric and body roundness indices associated with diabetes: Persian (Shahedieh) cohort study

**DOI:** 10.3389/fnut.2024.1428704

**Published:** 2024-08-12

**Authors:** Farnoosh Ghomi, Reyhane Sefidkar, Elham Khaledi, Sara Jambarsang

**Affiliations:** ^1^Student Research Committee, Department of Biostatistics and Epidemiology, School of Public Health, Shahid Sadoughi University of Medical Sciences, Yazd, Iran; ^2^Center for Healthcare Data Modeling, Departments of Biostatistics and Epidemiology, School of public health, Shahid Sadoughi University of Medical Sciences, Yazd, Iran

**Keywords:** type 2 diabetes, anthropometric indices, body roundness indices, cohort, incidence

## Abstract

**Introduction:**

Diabetes is a chronic and concerning health condition that poses a significant public health challenge. Given that preventing, detecting early, and treating T2DM can enhance public health outcomes, the objective of this study was to identify the most effective obesity indices and determine their optimal cut-off points for predicting the risk of T2DM in an Iranian population.

**Methods:**

This study was conducted on 8,019 male and female participants aged between 35 and 70 years in the context of Shahedieh cohort study. The ROC curve analysis was utilized to determine the optimal cut-off point of each anthropometric index to predict diabetes in age-sex categories.

**Results:**

The overall diabetes incidence in the study population was 2.5%, with 2.5% in men and 2.4% in women. In men, significant differences in most of the anthropometric indices were observed between diabetic individuals and healthy counterparts. This study found that for women 45–65, BMI and weight, and for men under 65 years, weight, WHR, BMI, WC, WHTR, AVI, and BRI are efficient T2DM predictors. The AUC of these indices varied from 0.593 (95% CI: 0.510–0.676) to 0.668 (95% CI: 0.586–0.750) in men, and from 0.587 (95% CI: 0.510–0.664) to 0.644 (95% CI: 0.535–0.754) in women.

**Conclusion:**

Anthropometric indices and body roundness are simple, inexpensive, and noninvasive means markers to predict the risk of diabetes. Our findings show that most of the studied indices had acceptable prediction power for men except for elderly. For women over 45 years old, weight and BMI are appropriate predictors. It seems that the approach of reducing diabetes incidence through early detection and primary prevention is achievable.

## 1 Introduction

Diabetes is one of the chronic and worrying diseases for public health. Examining the prevalence and incidence of diabetes is on the agenda of the World Health Organization because it can cause serious damage to body organs such as kidneys, heart, eyes, and vascular system (1). According to the latest predictions made by the International Diabetes Federation (IDF), approximately 463 million adults (9.3%) were diagnosed with diabetes in 2019, and it is also predicted that by 2045, approximately 700 (10.9%) million people will have diabetes (2). Diabetes and its complications will increase the global healthcare burden and mortality rates in the world. As a multifactorial disease, diabetes is caused by factors such as genetic factors and lifestyle (3).

The pathogenic factors of obesity refer to the pathogenesis of diabetes caused by obesity (4). Obesity is a known risk factor for type 2 diabetes, and the mechanism of this relationship depends on many factors, including people’s lifestyle, and its investigation is very complicated (5). Obesity leads to an increase in insulin resistance, and in this case, body cells respond less to insulin. Insulin is a hormone that is responsible for regulating blood sugar levels by facilitating the absorption of glucose into cells. When insulin resistance occurs, the body needs to produce more insulin to compensate, leading to a condition called hyperinsulinemia. Over time, the increased demand for insulin can reduce the ability of the pancreatic beta cells that are responsible for its production. This issue can lead to a decrease in insulin secretion, resulting in impaired glucose tolerance, and ultimately, T2DM. Hyperinsulinemia can cause symptoms such as obesity, fatigue, mood changes, excessive sweating, palpitations, and increased blood sugar levels (6–8). The pathogenesis of diabetes caused by obesity studies the relationship between obesity and the occurrence of diabetes by using anthropometric indicators (4). Anthropometric indices include weight, height, waist circumference (WC), body mass index (BMI), wrist circumference, and hip circumference.

Studies have shown that increasing BMI and WC are associated with an increased risk of type 2 diabetes (9). Even though BMI is one of the most common measures for general obesity, it cannot accurately reflect the location of fat in the body (3, 10). So, in the present study, several other anthropometric indices, which are more suitable indices for expressing obesity have been used.

Anthropometric indicators are among the most suitable pre-screening tools, because by using these indicators, it is possible to identify obese people and prevent the occurrence of diabetes in a non-invasive, easy, very reliable, scientific, and cost-effective way (11, 12). In this study, we want to investigate the power of weight, height, WC, BMI, wrist circumference, hip circumference, A Body Shape Index (ABSI), Conicity index, Waist-to-Hip Ratio (WHR), Waist-to-Height Ratio (WHTR), body roundness index (BRI), Body Adiposity Index (BAI), and Abdominal Volume Index (AVI) to predict the incidence of diabetes in an Iranian community. We also recognize the best anthropometric indices and detect the optimal cut-off point for each of these indices by age-sex grouping to predict diabetes incidence using the receiver operating characteristic (ROC) curve method.

## 2 Materials and methods

### 2.1 Study design and study population

The Shahedieh cohort study was conducted with the aim of investigating non-communicable diseases and their risk factors over 35 years old in Shahedieh city of Yazd. The Shahedieh cohort study is a population-based study and is part of the prospective Persian cohort study that was conducted in 18 regions of Iran (13). The date of enrollment started in 2016 (3). Demographic information, behavioral information, and lifestyle details were collected from all participants in the study through valid questionnaires. Anthropometric indexes, blood pressure, and laboratory variables were measured for all people and blood samples were taken from eligible people. During this 4-year follow-up period in the cohort study, the participants were contacted annually by phone and completed follow-up questionnaires regarding the occurrence of death or incidence of type 2 diabetes mellitus (T2DM). If a participant was diagnosed with T2DM, the researchers conducted further follow-up by visiting the home or hospital. They collected the participant’s medical records for evaluation and complete data recording, and if needed, medical examinations were performed to determine the diagnosis of T2DM (14).

### 2.2 Data collection, criteria and measurements

**Figure 1:**
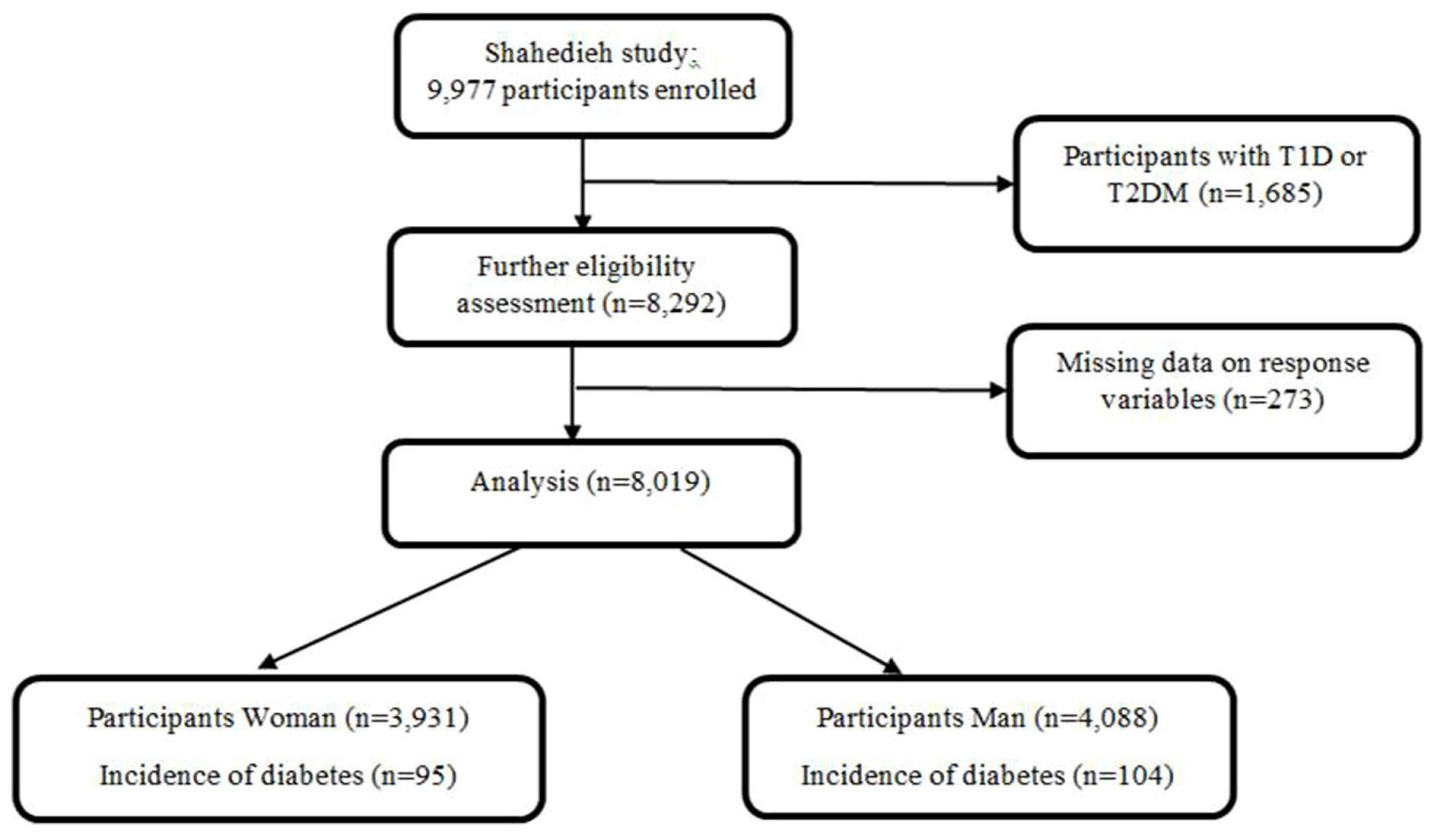
Diagram showing the selection process of participants from cohort.

Of the total 9,977 people who entered the study, 1,685 had diabetes in the enrollment phase and were excluded. 273 individuals were also excluded from the study due to pregnancy, incomplete anthropometric information, and diabetes status. The analysis was subsequently conducted on 8,019 men and women (Figure 1).

**Figure 2:**
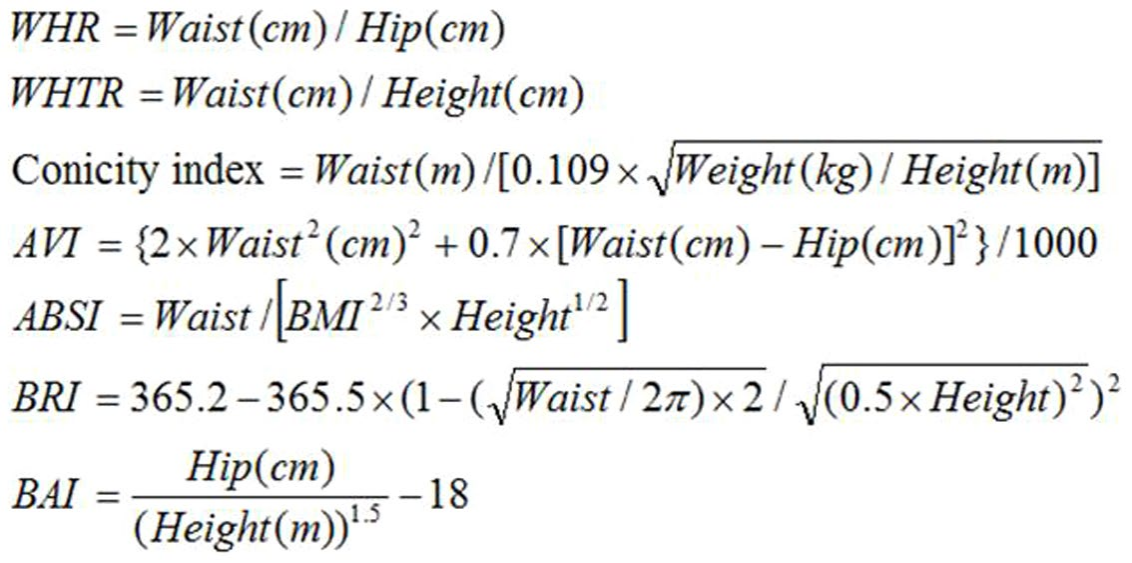
Anthropometric indices formulas.

People’s information was collected using questionnaires, clinical examinations, blood tests, and paraclinical tests. All anthropometric data were measured by trained health technicians and with digital scales and accurate instruments. The height of the participants was measured with bare feet in a state where their heads, shoulders, and heels were leaning against the wall, using a measuring tape attached to the wall with an accuracy of 0.5 cm. Their weight was measured using an Omron BF511 portable digital scale with an accuracy of 0.1 kg, in a standing position and with the smallest amount of clothing (14). BMI was calculated by dividing weight (kg) by the square of height (meters). WC was measured standing and immediately above the highest lateral border of the right pelvic ilium. Hip circumference was also measured while standing and placing the meter in the widest and most prominent part of the hip. People who had fasting blood sugar higher than 126 mg/dL or take medication were defined as people with diabetes. If the person was diagnosed with diabetes in the annual follow-up, further examination was done by visiting at home or hospital, and the medical documents related to the person were collected to evaluate. Medical examinations were performed to confirm the diagnosis of T2DM if it was necessary. The anthropometric and body roundness indices which were used in this study include BMI, height (cm), weight (kg), Waist circumference (cm), WC (cm), hip circumference (cm), WHR, WHTR, ABSI, Conicity index, BRI, AVI, BAI. Anthropometric indices and body roundness index WHTR, WHR, Conicity index, ABSI, AVI, BAI and BRI of people were calculated using the proposed formulas (Figure 2) (11, 15–21). Age was classified into 4 categories (35–45, 45–55, 55–65, and older than 65 years).

### 2.3 Statistical analysis of data

All anthropometric indices compared between T2DM and healthy individuals were analyzed using an independent t-test with SPSS version 26 software. A *p*-value less than 0.05 was considered significant. Cut-off points, AUC values, their confidence intervals (CI), sensitivity (se), specificity (sp), positive predictive value (PPV), and negative predictive value (NPV) were estimated for all anthropometric variables in age-sex groups using the “Optimal Cut points” package in R software (version 4.2.1).

## 3 Results

The study involved 8,019 participants with a mean age of 48.32 years [Standard Deviation (SD) = 9.54]. Of these participants, 51% were male. The age distribution was as follows: 44.8% were between 35 and 45 years old, 30.5% between 45 and 55 years old, 19.7% between 55 and 65 years old, and 5% were over 65 years old.

The incidence of diabetes was found to be 2.5% in the entire population, with 2.5% among men and 2.4% among women. Among men with diabetes, significant differences (*p* < 0.001) were observed in various anthropometric indices such as WC, BMI, WHR, WHtR, CI, AVI, BAI, and BRI, as well as in wrist circumference (*p* = 0.001), hip circumference (*p* = 0.002), and ABSI (0.015) compared to healthy participants. In contrast, among women, only wrist circumference (*p* = 0.02) was higher in those with diabetes compared to their healthy counterparts (Table 1).

**Table 1:** The comparison of baseline anthropometric indices means between T2DM and healthy individuals.

	Men			Women		
	Participants with T2DM incidence *N* = 104	Participants without T2DM *N* = 3,984		Participants with T2DM incidence *N* = 95	Participants without T2DM incidence *N* = 3,836	
	Mean (SD)	Mean (SD)	*p*	Mean (SD)	Mean (SD)	*p*
Weight (kg)	83.40 (15.53)	78.97 (13.86)	0.001	74.54 (11.79)	72.45 (13.12)	0.124
Waist (cm)	99.43 (12.58)	94.54 (11.6)	0.001≥	99.00 (10.82)	97.53 (11.88)	0.232
Hip (cm)	102.55 (8.50)	100.19 (7.58)	0.002	106.50 (9.25)	105.85 (10.02)	0.533
BMI	28.80 (4.74)	27.10 (4.25)	0.001≥	30.47 (4.23)	29.72 (5.12)	0.159
WHR	0.96 (0.06)	0.94 (0.06)	0.001≥	0.93 (0.06)	0.92 (0.07)	0.289
WHTR	0.58 (0.07)	0.55 (0.06)	0.001≥	0.63 (0.07)	0.62 (0.07)	0.307
Wrist	18.58 (1.37)	18.17 (1.25)	0.001	16.95 (1.33)	16.64 (1.25)	0.029
ABSI	0.08 (0.0034)	0.08 (0.0037)	0.015	0.08 (0.0053)	0.08 (0.0054)	0.550
Conicity index	1.30 (0.0692)	1.27 (0.0696)	0.001≥	1.318 (0.089)	1.316 (0.09)	0.890
AVI	20.12 (5.34)	18.16 (4.20)	0.001≥	19.91 (4.29)	19.39 (4.65)	0.288
BAI	28.33 (3.80)	27.04 (3.65)	0.001≥	36.58 (5.06)	36.37 (5.70)	0.721
BRI	5.20 (1.71)	4.53 (1.38)	0.001≥	6.32 (1.75)	6.15 (1.92)	0.376

According to Tables 2, 3, among men aged 35–45 years, WC (cut-off point 97 cm, AUC = 0.619; 95% CI: [0.536–0.702]),BMI (cut-off point 26.88 kg/m^2^, AUC = 0.610; 95% CI: [0.536–0.685]), WHR (cut-off point 0.948, AUC = 0.609; 95% CI: [0.529–0.688]), WHTR (cut-off point 0.541, AUC = 0.620; 95% CI: [0.543–0.698]), and wrist circumference (cut-off point 18.10 cm, AUC = 0.605; 95% CI: [0.525–0.685]) demonstrated the highest predictive power among anthropometric indices for type 2 diabetes mellitus (T2DM). Additionally, conicity index (cut-off point 1.284, AUC = 0.602; 95% CI: [0.519–0.684]), AVI (cut-off point 18.84, AUC = 0.619; 95% CI: [0.537–0.702]), BAI (cut-off point 25.36, AUC = 0.597; 95% CI: [0.517–0.677]), and BRI (cut-off point 4.18, AUC = 0.620; 95% CI: [0.543–0.698]) were the most effective body roundness indices for predicting T2DM incidence in this age group.

**Table 2:** Cut-off points, AUC, and confidence intervals for anthropometric parameters in men and women in 4 different age groups.

Men (*n* = 4,088)																
Age groups	35–45				45–55				55–65				>65			
	AUC	95% CI	se	sp	AUC	95% CI	se	sp	AUC	95% CI	se	sp	AUC	95% CI	se	sp
Weight (kg)	**0.593**	**(0.510, 0.676)**	**0.60**	**0.571**	**0.646**	**(0.564, 0.728)**	**0.515**	**0.701**	**0.597**	**(0.500, 0.695)**	**0.681**	**0.518**	0.495	(0.233, 0.757)	0.999	0.056
Waist (cm)	**0.619**	**(0.536, 0.702)**	**0.622**	**0.583**	**0.658**	**(0.577, 0.739)**	**0.787**	**0.465**	**0.639**	**(0.547, 0.731)**	**0.818**	**0.470**	0.509	(0.303, 0.715)	0.999	0.184
Hip (cm)	0.601	(0.514, 0.688)	0.55	0.64	**0.633**	**(0.550, 0.716)**	**0.72**	**0.42**	0.581	(0.481, 0.681)	0.50	0.70	0.440	(0.218, 0.663)	0.999	0.02
BMI	**0.610**	**(0.536, 0.685)**	**0.755**	**0.495**	**0.668**	**(0.586, 0.750)**	**0.575**	**0.725**	**0.633**	**(0.534, 0.733)**	**0.818**	**0.475**	0.489	(0.224, 0.755)	0.250	0.912
WHR	**0.609**	**(0.529, 0.688)**	**0.666**	**0.557**	**0.621**	**(0.535, 0.707)**	**0.757**	**0.497**	**0.646**	**(0.543, 0.750)**	**0.636**	**0.689**	0.651	(0.466, 0.836)	0.999	0.625
WHTR	**0.620**	**(0.543, 0.698)**	**0.80**	**0.429**	**0.660**	**(0.579, 0.742)**	**0.545**	**0.687**	**0.650**	**(0.555, 0.746)**	**0.727**	**0.615**	0.511	(0.278, 0.745)	0.999	0.235
Wrist	**0.605**	**(0.525, 0.68)**	**0.755**	**0.498**	**0.646**	**(0.552, 0.740)**	**0.818**	**0.417**	0.583	(0.435, 0.730)	0.545	0.733	0.307	(−0.062, 0.676)	0.250	0.835
Conicity index	**0.602**	**(0.519, 0.684)**	**0.622**	**0.565**	**0.608**	**(0.521, 0.695)**	**0.848**	**0.409**	0.587	(0.469, 0.706)	0.50	0.690	**0.786**	**(0.661, 0.911)**	**0.999**	**0.697**
ABSI	0.572	(0.488, 0.655)	0.488	0.643	0.572	(0.479, 0.664)	0.666	0.546	0.531	(0.406, 0.656)	0.50	0.686	**0.831**	**(0.701, 0.961)**	**0.999**	**0.692**
AVI	**0.619**	**(0.537, 0.702)**	**0.622**	**0.600**	**0.626**	**(0.534, 0.719)**	**0.787**	**0.489**	**0.644**	**(0.534, 0.754)**	**0.818**	**0.475**	0.507	(0.187, 0.827)	0.999	0.184
BAI	**0.597**	**(0.517, 0.677)**	**0.866**	**0.322**	**0.603**	**(0.506, 0.70)**	**0.727**	**0.517**	**0.628**	**(0.522, 0.734)**	**0.909**	**0.371**	0.538	(0.208, 0.869)	0.750	0.523
BRI	**0.620**	**(0.543, 0.698)**	**0.820**	**0.429**	**0.629**	**(0.534, 0.724)**	**0.545**	**0.687**	**0.663**	**(0.555, 0.772)**	**0.727**	**0.615**	0.586	(0.277, 0.895)	0.999	0.235

The numbers written in bold are the AUCs and confidence intervals of the anthropometric variables that are meaningful.

**Table 3:** The frequency and percentage of men who had anthropometric indices above the cut-off point, according to their sex and age group.

Men (*n* = 4,088)																
Age group (35–45) *N* = 1813					Age group (45–55) *N* = 1,282				Age group (55–65) *N* = 794				Age group (65–70) *N* = 199			
	Cut- off point	No. of subjects above cut-off point (%)	PPV	NPV	Cut-off point	No. of subjects above cut-off point (%)	PPV	NPV	Cut-off point	No. of subjects above cut-point (%)	PPV	NPV	Cut-off point	No. of subjects above cut-off point (%)	PPV	NPV
Weight (kg)	81	729 (40.21)	0.034	0.982	86	360 (20.08)	0.043	0.982	72	506 (63.73)	0.038	0.982	86	55 (27.63)	0.021	1.00
Waist (cm)	97	710 (39.16)	0.036	0.983	102	310 (24.18)	0.037	0.988	93.50	423 (53.27)	0.042	0.989	91.30	129 (64.82)	0.024	1.00
Hip (cm)	102.80	640 (35.30)	0.038	0.982	104.50	343 (26.75)	0.032	0.983	98.20	416 (52.39)	0.046	0.980	99.50	102 (51.26)	0.020	1.00
BMI	26.88	928 (51.18)	0.036	0.987	29.56	363 (28.31)	0.052	0.984	26.44	423 (53.27)	0.042	0.989	30.62	39 (19.60)	0.055	0.983
WHR	0.948	817 (45.06)	0.036	0.985	0.944	660 (51.48)	0.038	0.987	0.977	254 (31.98)	0.055	0.985	0.960	84 (42.21)	0.051	1.00
WHTR	0.541	1,060 (58.47)	0.034	0.988	0.587	410 (31.98)	0.044	0.982	0.568	318 (40.05)	0.051	0.987	0.512	160 (80.40)	0.026	1.00
Wrist	18.10	907 (50.03)	0.036	0.987	18	634 (49.45)	0.035	0.988	18.80	203 (25.57)	0.055	0.982	19.30	27 (13.57)	0.030	0.981
Conicity index	1.284	801 (44.18)	0. 35	0.983	1.262	765 (59.67)	0.036	0.990	1.312	250 (31.49)	0.044	0.979	1.317	63 (31.66)	0.063	1.00
ABSI	0.081	737 (9.2)	0.033	0.980	0.0806	592 (46.18)	0.037	0.984	0.082	285 (35.89)	0.043	0.979	0.083	65 (32.66)	0.062	1.00
AVI	18.84	735 (40.54)	0.038	0.984	17.74	664 (8.3)	0.039	0.988	17.54	424 (53.40)	0.042	0.989	14.79	163 (81.91)	0.024	1.00
BAI	25.36	1,240 (68.39)	0.031	0.989	27.02	627 (48.91)	0.038	0.986	25.48	505 (63.60)	0.039	0.993	27.29	96 (48.24)	0.031	0.990
BRI	4.18	1,051 (57.97)	0.0344	0.988	5.15	409 (31.90)	0.044	0.982	4.74	313 (39.42)	0.051	0.987	3.69	153 (76.88)	0.026	1.00

In men aged 45–55 years, all indices were more effective predictors of T2DM compared to those aged 35–45 years. For men aged 55–65 years, weight (cut-off point 72 kg, AUC = 0.597; 95% CI: [0.500–0.695]), WC (cut-off point 93.5 cm, AUC = 0.639; 95% CI: [0.547–0.731]), BMI (cut-off point 26.44 kg/m^2^, AUC = 0.633; 95% CI: [0.534–0.733]), WHR (cut-off point 0.977, AUC = 0.646; 95% CI: [0.543–0.750]), and WHTR (cut-off point 0.568, AUC = 0.650; 95% CI: [0.555–0.746]) were significant anthropometric indices for predicting T2DM incidence. In the same age group, AVI (cut-off point 17.54, AUC = 0.644; 95% CI: [0.534–0.754]), BAI (cut-off point 25.48, AUC = 0.628; 95% CI: [0.522–0.734]), and BRI (cut-off point 4.74, AUC = 0.663; 95% CI: [0.555–0.772]) were useful body roundness indices for prediction.

In women aged 35–45 years, none of the anthropometric indices predicted the incidence of diabetes. For women aged 45–55 years, weight (cut-off point 79 kg, AUC = 0.588; 95% CI: [0.508–0.667]) and BMI (cut-off point 26.48 kg/m^2^, AUC = 0.587; 95% CI: [0.510–0.664]) were effective predictors of T2DM. Among women aged 55–65 years, weight (cut-off point 77 kg, AUC = 0.644; 95% CI: [0.535–0.754]) was predictive, while for those over 65 years, hip circumference (cut-off point 106 cm, AUC = 0.655; 95% CI: [0.506–0.805]), BMI (cut-off point 28.53 kg/m^2^, AUC = 0.656; 95% CI: [0.517–0.795]), BAI (cut-off point 40.41, AUC = 0.794; 95% CI: [0.681–0.907]), and BRI (cut-off point 6.06, AUC = 0.652; 95% CI: [0.532–0.773]) showed acceptable predictive power for T2DM incidence. Detailed specificity, sensitivity, PPV, and NPV values are provided in Tables 4, 5.

**Table 4:** Cut-off points, AUC, sensitivity, specificity, and confidence intervals for anthropometric parameters in women in 4 different age groups.

Women (*n* = 3,931)																
Age groups	35–45				45–55				55–65				>65			
	AUC	95% CI	se	sp	AUC	95% CI	se	sp	AUC	95% CI	se	sp	AUC	95% CI	se	sp
Weight (kg)	0.531	(0.454, 0.607)	0.818	0.283	**0.588**	**(0.508, 0.667)**	**0.933**	**0.233**	**0.644**	**(0.535, 0.754)**	0.933	0.367	0.570	(0.381, 0.759)	0.99	0.226
Waist (cm)	0.515	(0.435, 0.594)	0.886	0.197	0.566	(0.488,0.644)	0.833	0.314	0.606	(0.476,0.735)	0.733	0.516	0.566	(0.376, 0.755)	0.666	0.623
Hip (cm)	0.467	(0.392, 0.542)	0.93	0.15	0.570	(0.483,0.656)	0.40	0.73	0.584	(0.450,0.717)	0.46	0.72	**0.655**	**(0.506, 0.805)**	**0.83**	**0.59**
BMI	0.521	(0.446, 0.597)	0.909	0.187	**0.587**	**(0.510, 0.664)**	**0.933**	**0.283**	0.610	(0.479,0.740)	0.466	0.688	**0.656**	**(0.517, 0.795)**	**0.99**	**0.432**
WHR	0.563	(0.484, 0.641)	0.704	0.385	0.544	(0.458, 0.630)	0.60	0.486	0.554	(0.431, 0.676)	0.733	0.626	0.401	(0.252, 0.550)	0.99	0.175
WHTR	0.506	(0.428, 0.584)	0.386	0.702	0.558	(0.479, 0.636)	0.900	0.235	0.579	(0.445, 0.713)	0.266	0.919	0.613	(0.483, 0.742)	0.99	0.477
Wrist	0.550	(0.464, 0.636)	0.295	0.816	0.595	(0.497, 0.693)	0.733	0.444	0.610	(0.464, 0.757)	0.533	0.690	0.559	(0.304, 0.813)	0.333	0.854
Conicity index	0.484	(0.391, 0.578)	0.181	0.866	0.505	(0.405, 0.605)	0.70	0.378	0.567	(0.402, 0.731)	0.466	0.771	0.482	(0.272, 0.691)	0.99	0.236
ABSI	0.479	(0.383, 0.576)	0.431	0.644	0.483	(0.388, 0.578)	0.666	0.459	0.53	(0.377, 0.683)	0.333	0.798	0.408	(0.168, 0.648)	0.833	0.251
AVI	0.513	(0.427, 0.6)	0.318	0.776	0.538	(0.444, 0.632)	0.833	0.315	0.596	(0.439, 0.754)	0.733	0.520	0.596	(0.383, 0.809)	0.666	0.638
BAI	0.479	(0.394, 0.565)	0.545	0.529	0.565	(0.472, 0.658)	0.866	0.30	0.504	(0.327, 0.68)	0.466	0.687	**0.794**	**(0.681, 0.907)**	**0.833**	**0.768**
BRI	0.501	(0.416, 0.586)	0.386	0.702	0.536	(0.442, 0.630)	0.90	0.235	0.566	(0.399, 0.734)	0.266	0.919	**0.652**	**(0.532, 0.773)**	**0.99**	**0.477**

The numbers written in bold are the AUCs and confidence intervals of the anthropometric variables that are meaningful.

**Table 5:** The frequency and percentage of women who had anthropometric indices above the cut-off point, according to their sex and age group.

Women (*n* = 3,931)																
Age group (35–45) *N* = 1780					Age group (45–55) *N* = 1,160				Age group (55–65) *N* = 786				Age group (65–70) *N* = 205			
	Cut-off point	No. of subjects above cut-off point (%)	PPV	NPV	Cut-off point	No. of subjects above cut-off point (%)	PPV	NPV	Cut-off point	No. of subjects above cut-off point (%)	PPV	NPV	Cut-off point	No. of subjects above cut-off point (%)	PPV	NPV
Weight (kg)	66	1,224 (68.76)	0.028	0.984	79	295 (25.43)	0.031	0.03	77	243 (30.91)	0.02	0.99	73	85 (41.46)	0.03	1.00
Waist (cm)	88	1,398 (78.54)	0.272	0.985	91.40	799 (68.88)	0.031	0.986	99	370 (47.07)	0.028	0.990	90	154 (75.12)	0.050	0.984
Hip (cm)	97	1,474 (82.81)	0.272	0.989	107	456 (39.31)	0.038	0.978	110	206 (26.21)	0.031	0.985	106	96 (46.83)	0.058	0.991
BMI	25.61	1,451 (81.52)	0.275	0.987	26.48	838 (72.24)	0.033	0.993	31.63	247 (31.42)	0.028	0.985	28.53	119 (58.05)	0.050	1.00
WHR	0.898	1,110 (62.36)	0.028	0.980	0.910	706 (60.86)	0.030	0.979	0.953	301 (38.29)	0.036	0.991	0.851	170 (82.93)	0.035	1.00
WHTR	0.662	537 (30.17)	0.031	0.978	0.566	897 (77.32)	0.030	0.988	0.661	272 (34.60)	0.060	0.984	0.604	137 (66.83)	0.054	1.00
Wrist	17.80	313 (17.58)	0.039	0.978	16.40	636 (54.83)	0.033	0.984	17.20	231 (29.39)	0.032	0.987	18	23 (11.22)	0.064	0.977
Conicity index	1.412	242 (13.59)	0.033	0.976	1.285	724 (62.41)	0.029	0.979	1.393	184 (23.41)	0.038	0.986	1.259	158 (77.07)	0.037	1.00
ABSI	0.083	737 (41.40)	0.029	0.978	0.0811	636 (54.83)	0.031	0.981	0.086	200 (25.44)	0.038	0.984	0.078	157 (76.58)	0.032	0.980
AVI	22.55	402 (22.58)	0.034	0.978	16.79	799 (68.88)	0.031	0.986	19.60	385 (48.98)	0.028	0.990	20.45	76 (37.07)	0.052	0.984
BAI	35.89	844 (47.41)	0.028	0.978	32.94	817 (70.43)	0.031	0.988	38.34	248 (31.55)	0.028	0.985	40.41	51 (24.88)	0.098	0.993
BRI	6.92	536 (30.11)	0.031	0.978	4.72	893 (76.98)	0.030	0.988	8.87	67 (9.52)	0.060	0.984	6.06	110 (53.66)	0.054	1.00

All the ROC curves for entire anthropometric and body roundness indices by age-sex categories are in the Supplementary material.

## 4 Discussion

The primary aim of this study was to assess the incidence of T2DM and its related obesity factors optimal cut-off points to predict it among Iranian adults. This research revealed that the incidence of T2DM was slender. Beigrezaei et al. showed that about 2.8% of adults in Yazd, Iran have been affected by T2DM. In another study which was done in Turkey, the incidence of T2DM was 2.9% (22).

In our study, for men, most of the anthropometric and body roundness indices were known as useful screening tools to predict the incidence of T2DM in participants between 35 and 55 years old, especially in 45–55 age group. The most predictive powers were related to WHTR and BMI in 35–45 and 45–55 age groups, respectively. In men between 55 and 65 years, most of the anthropometric and roundness indices had acceptable power except wrist circumference, hip circumference, ABSI and Conicity index. Among these indices, BRI predicted T2DM effectively in this age group. For men over 65 years, Conicity index and ABSI had the highest AUCs. In our study, in 35–45 years old men, the estimated optimal cut-off point for WHTR to predict T2DM was 0.541 which was almost different to those studies. In 45–55 year old men 29.56 kg/m^2^ and in 55–65 year old men 4.74 were the estimated optimal cut-off point for BMI and BRI to predict T2DM, respectively.

In women, most of the anthropometric and body roundness indices did not predict T2DM efficiently. In 45–55 year old women, weight and BMI were adequate predictors with optimal cut-off points 79 kg and 26.48 kg/m^2^, respectively. Weight had also an effective power to predict T2DM with estimated cut-off point 77 kg in 55–65 year old women. Among the studied indices, BAI with calculated cut-off point 6.06 has the best AUC in women over 65 years. Based on literature some of the anthropometric indices are poor predictor of abdominal visceral fat (AVF) in women, such as waist-to-hip ratio and its useable as a surrogate measure of visceral fat (23, 24). Based on the meta-analysis results, AVF is associated with an increased risk of T2DM. Failure to observe a significant relationship between anthropometric indices and diabetes in women can be caused by this lack of measurement of visceral fat. This can be a confirmation of the recommendation to pay attention to the higher chance of diabetes in apparently thin people with high visceral obesity (24).

In a case control study which was conducted in India, BMI and WC were identified to have good sensitivity and specificity in both sexes irrespective of age. Cut-off point of BMI and WC were 22.07 kg/m^2^ and 91.25 cm for males and 22.28 kg/m^2^ and 83.5 cm for females, respectively (9). In a longitudinal study on 2,810 Iranian females over a median follow up of 3.5 years, 4.1% of individuals developed diabetes (4.1%). BMI, WC, WHR, WHTR were predictive of development of type 2 diabetes, but WHTR was a better predictor than BMI (25). Based on a systematic review of longitudinal studies, a strong association between measures reflecting abdominal obesity and the development of T2DM was revealed (26). According to a cross sectional study on middle-aged and elderly Chinese, WHTR and WHR have been found to perform better predictors of T2DM compared to other obesity anthropometric indices for males and females, respectively (27). In another study on north-east Chinese adults, it was reported that the Maximal BMI in the past (MAXBMI) was a good anthropometric index associated with T2DM (28). Individuals with MAXBMI over 23 kg/m^2^ are prone to diabetes. In a cross-sectional study on data from the National basic health research of Indonesia in 2013, it was found that WTHR has better predictive power than BMI and waist circumference with optimal cut-off points 0.48 and 0.53 for men and women, respectively (29). Based on a result of a retrospective longitudinal study, both BMI and WC indicated a positive association with diabetes in both sexes. This research suggested that cut-off points of 25.1 kg/m2 for BMI and 88.0 cm for WC in men, as well as 24.4 kg/m2 for BMI and 78.2 cm for WC in women, may play a crucial role in developing effective strategies for diabetes prevention (30). In over 45-year-old women, the anthropometric and body roundness indices were significant D2MS predictors. This finding may due to this fact that postmenopausal women had two-fold higher concentrations of both visceral and subcutaneous adipose tissue than premenopausal women (31). Furthermore, the onset of post menopause which is characterized by a decline in estradiol levels, which can have a detrimental effect on the expression of genes involved in lipid metabolism (32). As a result of aging in men as well as women, there is a notable increase in the distribution of visceral fat mass, which coincides with a rise in waist circumference (WC). The findings of the current study showed that body mass index (BMI) in men has comparable predictive power for diabetes risk as WC which seems to contradict the finding of Makiko et al.’s study (30).

A healthy lifestyle, including regular physical activity, a balanced diet, and self-maintenance, can help to prevent the onset of insulin resistance, regardless of sex, although the different habits between men and women greatly affect the implementation of preventative guidelines that help in fighting the manifestations of this metabolic disorder (30). Based on the evidence obtained from a meta-analysis including 4,090 people, it is possible to develop T2DM with lifestyle changes including diet, weight loss, and increased physical activity. This meta-analysis of clinical trials showed that the risk reduction continues even years after weight loss interventions (33). The global crisis of T2DM threatens all countries’ economies, especially developing countries. Meanwhile, in Asian countries, diabetes develops at a lower level of BMI due to the high carbohydrate (white rice) lifestyle and diet (34). Therefore, it is important to pay attention to the lifestyle and keep the anthropometric indices at the appropriate level to prevent T2DM in these communities.

This study has robust statistical findings because it is based on a large, population-based sample from the central region of Iran. The effectiveness of obesity-related markers and their optimal cut-off points for predicting type 2 diabetes mellitus T2DM can vary across different races and ethnicities, so it is crucial to evaluate these indices locally. Furthermore, since the anthropometric indices were measured before the incidence of T2DM, the investigated relationships further give the concept of causality. This study has several limitations. Due to the small number of T2DM incidences in some age groups, the estimated cut-off points may be insupportable. Another limitation of this study was lack of neck circumference measurement in Shahedieh study, which is an important obesity indicator.

## 5 Conclusion

In conclusion, our findings showed that in women between 45 and 65 years BMI and weight and in men less than 65 years weight, WHR, BMI, WC, WHTR, AVI, and BRI are the most important indicators for T2DM incidence. Due to the T2DM pandemic in developing countries, these indices which are extremely effective, simple, inexpensive, and noninvasive means have a vital public health implication for first-level screening. Current study also has another important finding it can be remarked that choosing the best index for predicting T2DM locally.

## Data availability statement

The data analyzed in this study is subject to the following licenses/restrictions: the Shahedieh cohort study is among the Persian cohort studies launched in 21 provinces of Iran thus far. All this data has been registered nationally, and access to each cohort has been provided to the conducting center and university. If you have any questions about the data, you can ask the author. Requests to access these datasets should be directed to s.jambarsang@gmail.com.

## Ethics statement

The Shahedeh cohort study has ethics approval from the Ethics Committee of Shahid Sadoughi University of Medical Sciences, Yazd (IR.SSU.SPH.REC.1397.135). The objectives of the study, the confidentiality of information, and the method of conducting the study were explained to the participants, and they entered the study voluntarily. The study protocol was in accordance with the ethical principles of the Declaration of Helsinki. This study has been approved by the ethics committee of Yazd University of Medical Sciences with the code of ethics (IR.SSU.SPH.REC.1401.106). The studies were conducted in accordance with the local legislation and institutional requirements. Written informed consent for participation in this study was provided by the participants’ legal guardians/next of kin.

## Author contributions

FG: Data curation, Formal analysis, Methodology, Visualization, Writing – original draft, Writing – review & editing, Conceptualization, Investigation, Project administration, Resources, Software, Supervision, Validation. RS: Methodology, Project administration, Writing – review & editing, Writing – original draft, Formal analysis, Conceptualization, Data curation, Investigation, Resources, Software, Supervision, Validation, Visualization. EK: Formal analysis, Methodology, Writing – original draft, Supervision, Validation, Writing – review & editing, Conceptualization, Data curation, Investigation, Project administration, Resources, Software, Visualization. SJ: Formal analysis, Methodology, Supervision, Validation, Writing – original draft, Writing – review & editing.
