# Supplementary material for: Optimal cut-off points of anthropometric and body roundness indices associated with diabetes: Persian (Shahedieh) cohort study
Source: Front Nutr. 2024 Aug 12;11:1428704. doi: 10.3389/fnut.2024.1428704 (PMC11345168; doi:10.3389/fnut.2024.1428704)
Supplement: Supplementary file 1 [file Table_1.DOCX]

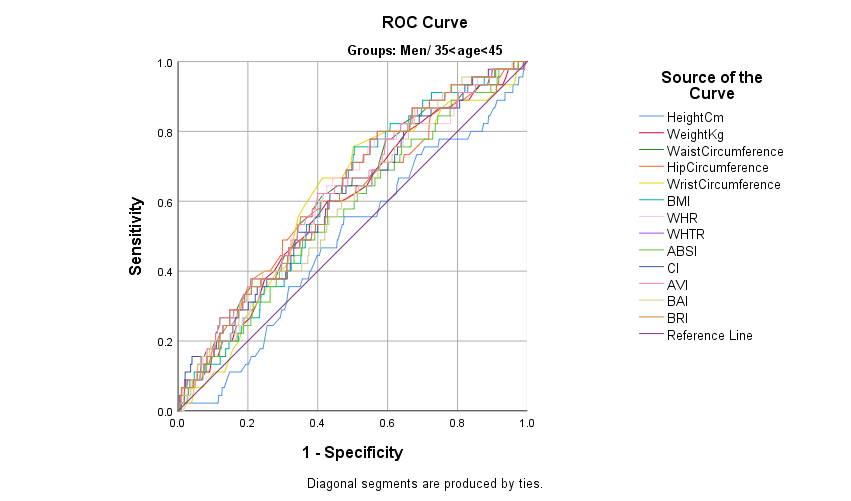


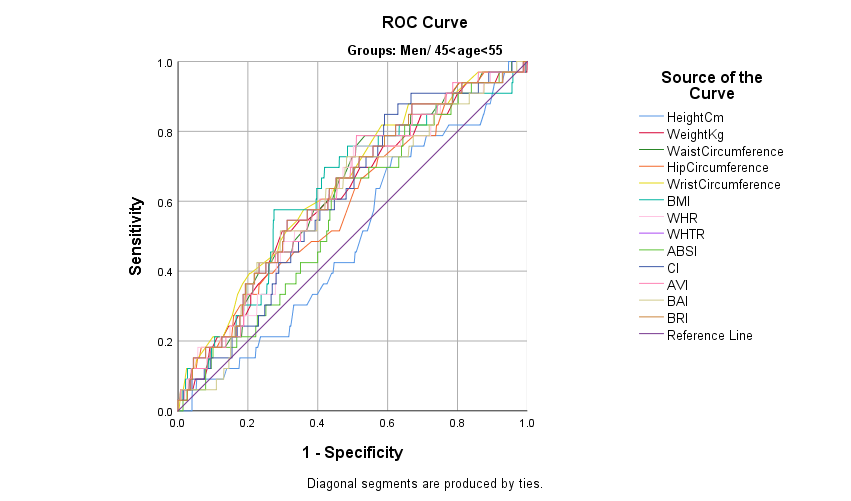


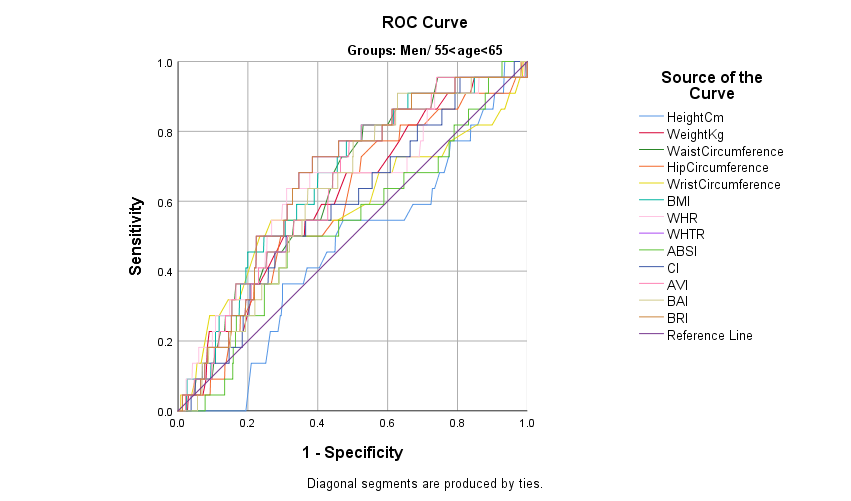

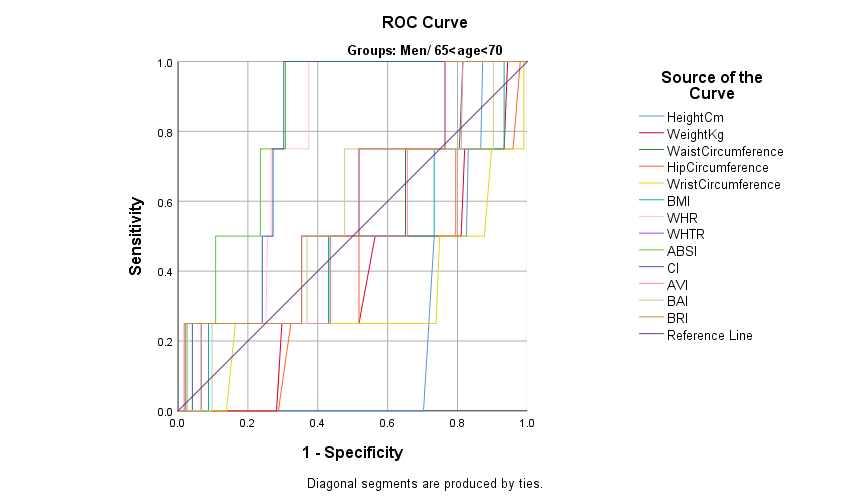


As depicted in ROC plots, for men in all four age groups, the largest area under the curve (AUC) was observed for the age range 65 to 70 years with the CI and ABSI indices. Different indicators were found to be significant and had varying AUCs across different age ranges, as detailed in the text of the article. The height index showed no significance in any age group and had no effect on the incidence of diabetes.


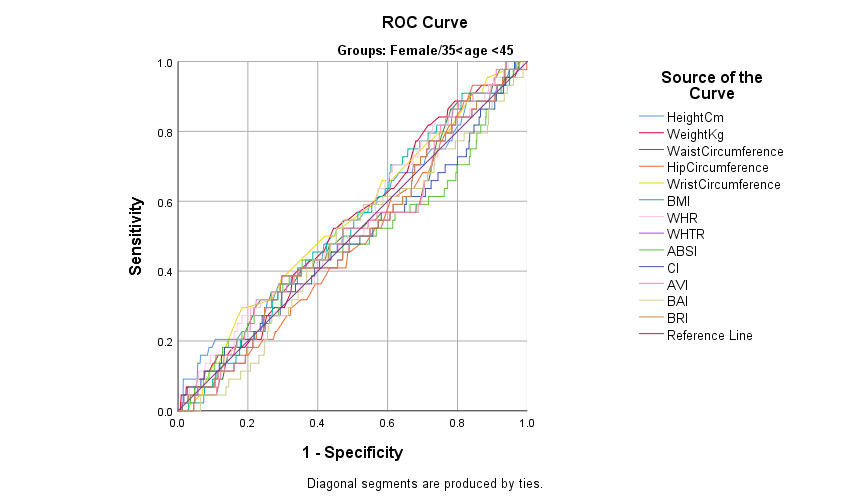

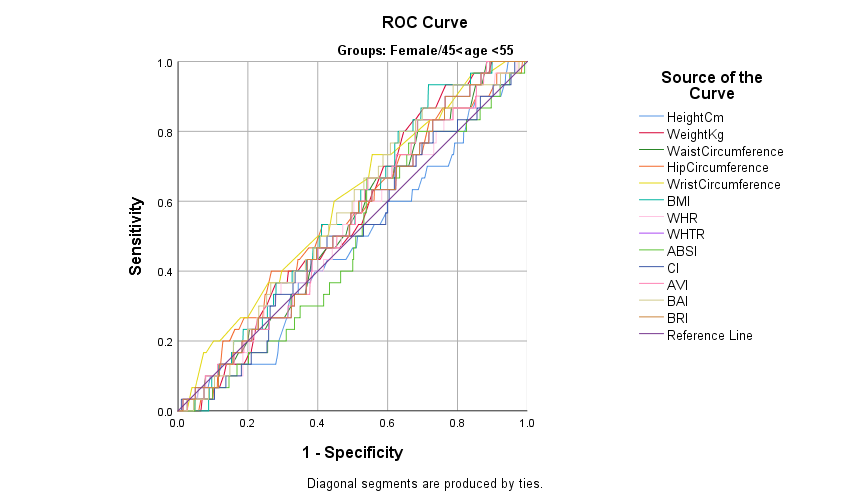


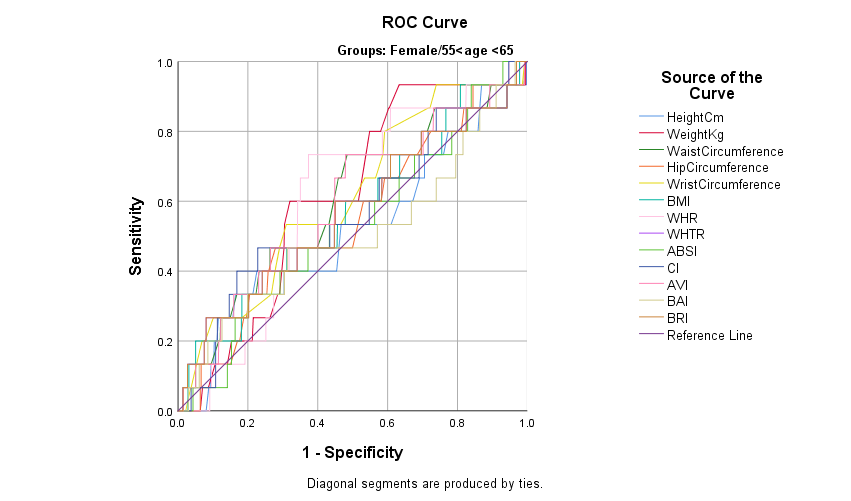

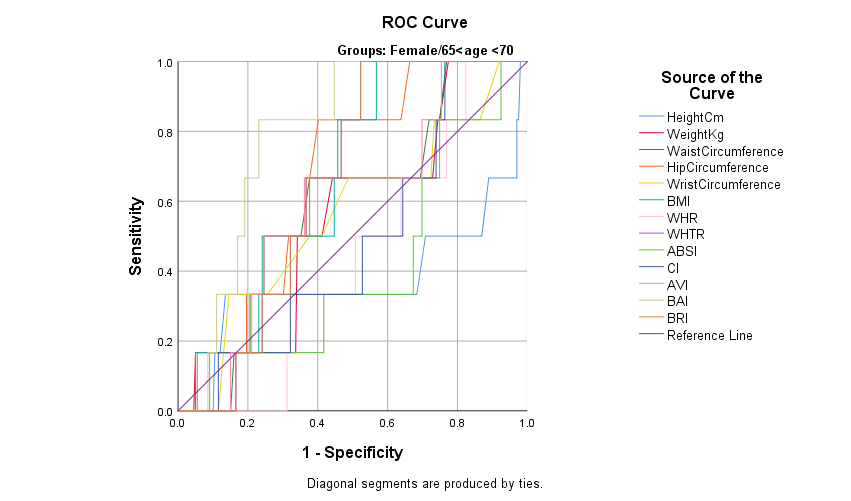


Since the ROC plots for women are also displayed in all four age ranges, the highest area under the curve (AUC) for BAI and BRI indices is in women with the age range of 65 to 70 years. Different indices were significant that showed different AUCs in different age ranges as explained in the text of the article.
